# Identifying early-measured variables associated with APACHE IVa providing incorrect in-hospital mortality predictions for critical care patients

**DOI:** 10.1038/s41598-021-01290-7

**Published:** 2021-11-12

**Authors:** Shuo Feng, Joel A. Dubin

**Affiliations:** 1 Department of Statistics and Actuarial Science, University of Waterloo, Waterloo, ON Canada; 2 School of Public Health Sciences, University of Waterloo, Waterloo, ON Canada

**Keywords:** Statistics, Medical research

## Abstract

APACHE IVa provides typically useful and accurate predictions on in-hospital mortality and length of stay for patients in critical care. However, there are factors which may preclude APACHE IVa from reaching its ceiling of predictive accuracy. Our primary aim was to determine which variables available within the first 24 h of a patient’s ICU stay may be indicative of the APACHE IVa scoring system making occasional but potentially illuminating errors in predicting in-hospital mortality. We utilized the publicly available multi-institutional ICU database, eICU, available since 2018, to identify a large observational cohort for our investigation. APACHE IVa scores are provided by eICU for each patient’s ICU stay. We used Lasso logistic regression in an aim to build parsimonious final models, using cross-validation to select the penalization parameter, separately for each of our two responses, i.e., errors, of interest, which are APACHE falsely predicting in-hospital death (Type I error), and APACHE falsely predicting in-hospital survival (Type II error). We then assessed the performance of the models with a random holdout validation sample. While the extremeness of the APACHE prediction led to dependable predictions for preventing either type of error, distinct variables were identified as being strongly associated with the two different types of errors occurring. These included a primary set of predictors consisting of mean SpO2 and worst lactate for predicting Type I errors, and worst albumin and mean heart rate for Type II. In addition, a secondary set of predictors including changes recorded in care limitations for the patient’s treatment plan, worst pH, whether cardiac arrest occurred at admission, and whether vasopressor was provided for predicting Type I error; age, whether the patient was ventilated in day 1, mean respiratory rate, worst lactate, worst blood urea nitrogen test, and mean aperiodic vitals for Type II. The two models also differed in their performance metrics in their holdout validation samples, in large part due to the lower prevalence of Type II errors compared to Type I. The eICU database was a good resource for evaluating our objective, and important recommendations are provided, particularly identifying key variables that could lead to APACHE prediction errors when APACHE scores are sufficiently low to predict in-hospital survival.

## Introduction

The acute physiology and and chronic health evaluation scoring system, otherwise known as APACHE, is widely known to be at least a reasonable, if not good, predictive tool for in-hospital mortality and length of stay for patients in critical care^1^. Its current iteration is APACHE IVa, a relatively minor but improved update to APACHE IV, which itself was a major update to the APACHE scoring system^2^. APACHE scores are generated from many demographic and physiologic measures, as well as diagnosis, collected during the first 24 h of a patient’s ICU stay^2^.

Despite its usefulness for predicting outcomes such as in-hospital mortality of ICU patients, APACHE (IVa) is not infallible. For example, it has been shown to be challenging to externally validate the promising results of predicting in-hospital mortality to different countries (e.g., in Iran^3^, and India^4^). Even in settings where it is generally accurate, such as for predicting in-hospital mortality in United States-based ICU’s, there are dynamics which may preclude APACHE from reaching its ceiling of predictive accuracy. For example, patients with high APACHE scores can receive sufficient intervention during their stay to avoid a predicted in-hospital death (i.e., a resulting *false positive*), and patients with low APACHE scores may experience unexpected complications during their stay, which may result in an in-hospital death that APACHE failed to predict (i.e., a *false negative*). In addition, before these types of dynamics can lead to an unexpected patient outcome, there may actually be features observed in the first 24 h of an ICU patient’s stay that are predictive of an inaccurate APACHE prediction.

Note that, in general, it is hard to argue that intervening on a high APACHE score is an incorrect choice. Without the counterfactual, we will not know if choosing not to intervene would have led to a similar outcome for those where the APACHE score leads to a false positive, i.e., a good outcome for the patient. However, one could argue that not intervening due to a low APACHE score (or at least not intervening as seriously as one might consider if the score was higher, especially when resources are limited) might be one of the reasons for a false negative prediction. Hence, false negatives are especially important to focus upon in terms of seeing patient features that are associated with them. This is also the primary motivation to separate the analysis of the two types of APACHE prediction errors.

In this paper, we aim to quantify both the number of false positive and false negatives of APACHE IVa in a large heterogeneous multi-hospital ICU database collected from over 200 hospitals in the United States^5^. In addition, we will present models that show which features collected within the first 24 h of the ICU stay are associated with each of these two types of APACHE prediction error. That is, we will identify variables early in an ICU stay that might be predictive of APACHE IVa not doing as well in its outcome prediction of in-hospital mortality as expected. We will defer to future work to describe the dynamics after the first 24 h of a patient’s ICU stay that may lead to the APACHE predictions not being correct.

The paper is organized as follows. In “Methods”, we describe the multi-hospital database we are utilizing for our current study, how the cohort for this paper was chosen, as well as present details on variable definitions and how missing data were handled. In addition, we discuss our quantitative methods and various decisions that were required before and during the modeling stage. In “Results”, we present our findings, and in “Discussion”, we discuss our findings and point to various areas of interesting future work.

## Methods

### Description of eICU database

The ICU database that we will be using in this paper is formally referred to as the *eICU Collaborative Research Database*, version 2.0 (PhysioNet^5^). There are over 200,000 de-identified ICU admissions in over 200 hospitals across the United States, during the period from 2014 to 2015. Further details on this database, including available patient measurements, diagnoses, care planning, etc., can be found in Pollard et al.^5^). We will discuss what aspects of the database that we will be using, as well as how we defined the final cohort used in our analyses, in the subsections below.

### APACHE scores

The APACHE scores provide a typically accurate estimate of ICU patients’ in-hospital mortality^2^. Patient APACHE IVa scores, and associated in-hospital mortality prediction probabilities, are both obtained directly from one of the available eICU tables, titled *apachePatientResult*. APACHE IVa tends to overpredict mortality risk, which, in our analysis cohort, produces an average mortality rate of 11.96% compared to the observed average mortality of 9.91%. Figure 2a,b summarizes the mortality prediction from APACHE versus the true mortality across the more than 200 different hospitals in the eICU database. Each point on Fig. 2a,b represents a hospital.

**Figure 1:**
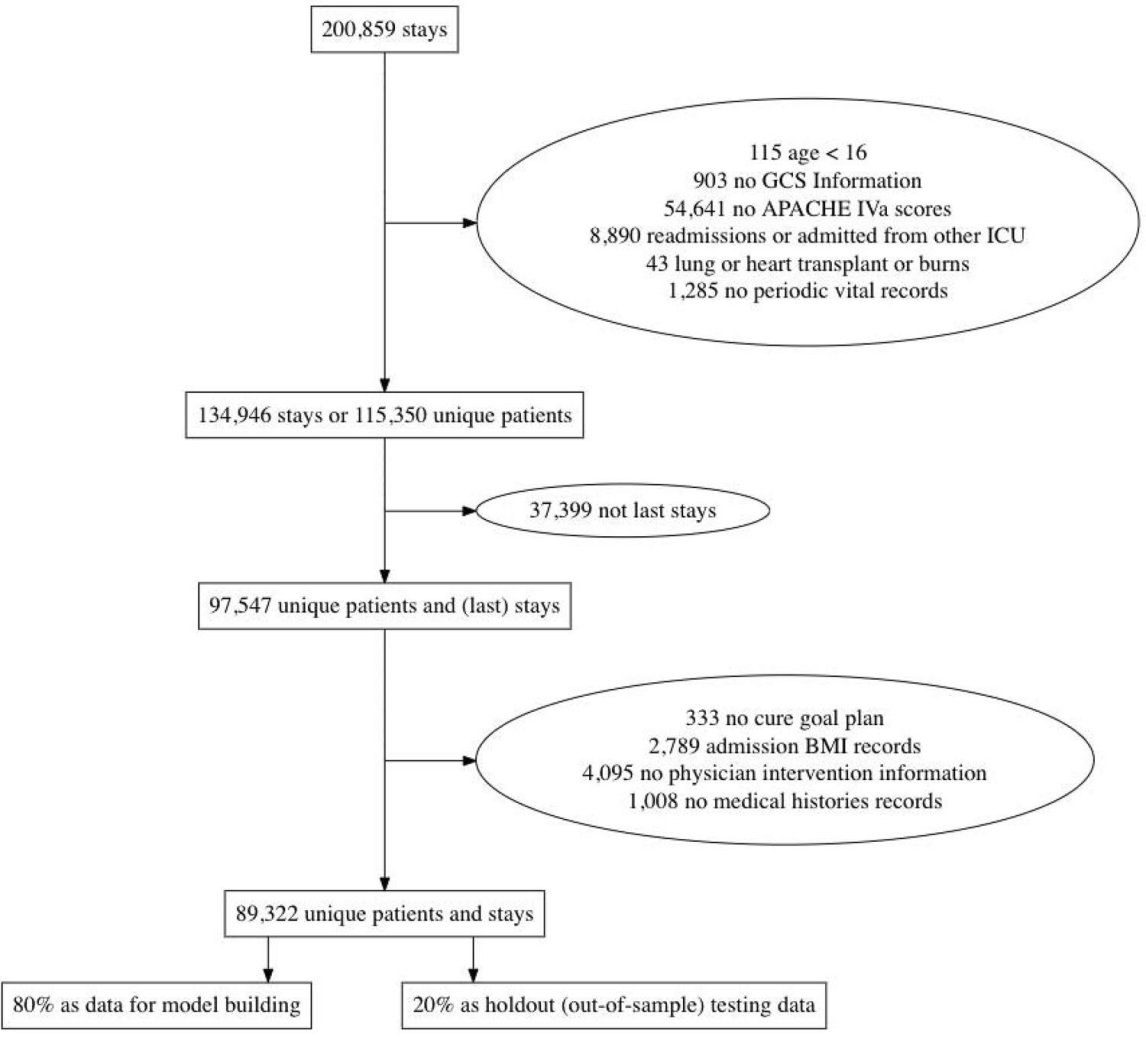
Flow chart of cohort selection from eICU database.

**Figure 2:**
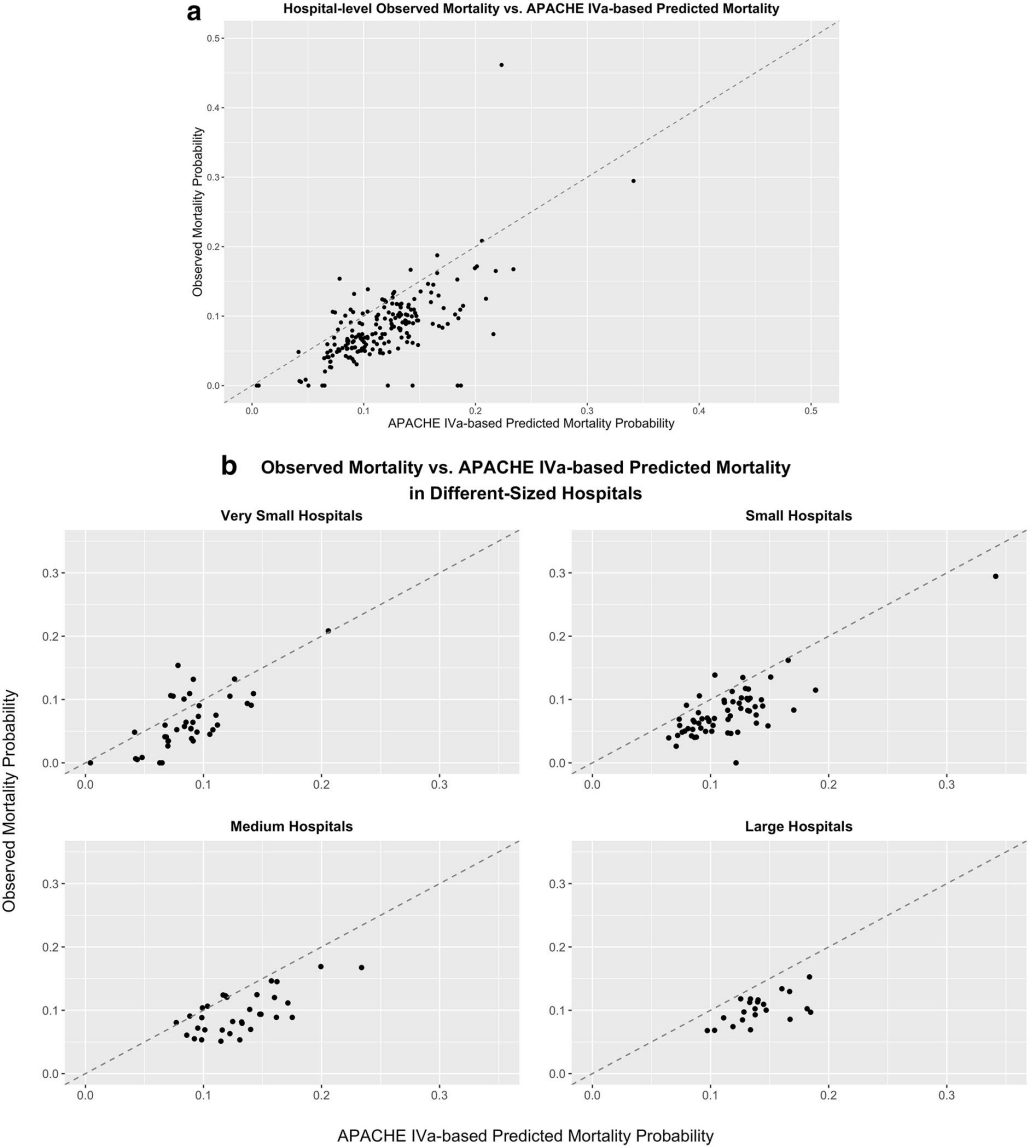
(**a**) Hospital-level observed mortality versus APACHE IVa-based predicted mortality. (**b**) Observed mortality versus APACHE IVa-based predicted mortality in different-sized hospitals.

For Fig. 2a, the points tend to cluster below the red dashed diagonal line, suggesting a higher predicted mortality from APACHE IVa than the actual mortality.

For Fig. 2b, we observed the same tendency for points to cluster below the red dash line among different-sized hospitals, i.e., very small (with < 100 beds), small (100–249 beds), medium (250–499 beds) and large hospitals ($\ge$ 500 beds).

In our subsequent analyses, our target responses are defined as the two types of errors that the APACHE IVa model could make:

1. *Type I Error*, where APACHE predicted a patient to die before hospital discharge but the patient survived the hospital stay. This represents a *false positive* in APACHE mortality predictions; and
2. *Type II Error*, where APACHE predicted a patient to survive to discharge but the patient died before being discharged alive from the hospital stay. This represents a *false negative* in APACHE mortality predictions.

### Variable definitions

We have incorporated the following six sources of variables into our analyses:

1. Vital sign values, which includes the mean and worst values of vital signs captured in the first 24 h of an ICU stay, as suggested by Cosgriff et al.^6^;
2. The most abnormal laboratory results;
3. Treatments;
4. Care plan and cure goals documented by physicians;
5. Admission diagnoses; and
6. Patients’ medical history.

Variables from sources 1, 2, 3, and 4 were measured throughout the entire in-hospital stay, but we only considered the values observed within the first 24 h of an ICU stay to align with the data used in the calculation of APACHE (IVa) scores.

Variable sets 1 and 2 were created in light of the definitions recommended by Cosgriff et al.^6^, i.e., we only considered the mean and worst values for vital signs, and the worst laboratory test results in the first 24 h. Specifically, we extracted the minimum values for base excess, calcium, chloride, fibrinogen, hematocrit, hemoglobin, magnesium, pH, phosphate, platelets, and serum bicarbonate, and the maximum values for amylase, B-natriuretic peptide, bilirubin, blood urea nitrogen, creatinine phosphokinase, lactate, lipase, prothrombin time, serum creatinine, and troponin. For glucose and serum sodium, we selected the value that deviated the most from its normal range. For neutrophil and white blood cell counts, the minimum value was selected only if any measurements fell lower than the lower limit of its normal range; otherwise, the maximum was selected.

In dealing with the admission diagnoses, which were available only in text form, Cosgriff et al.^6^ suggested a way to group them into 21 meaningful categories, i.e., acute coronary syndrome (ACS), acute renal failure (ARF), asthma emphys, coronary artery bypass grafting (CABG), cardiac arrest, chest pain, congestive heart failure (CHF), coma, cerebrovascular accident/stroke (CVA), diabetic ketoacidosis (DKA), gastrointestinal (GI) bleed, GI obstruction, neurologic problems, overdose, pneumonia (PNA), sepsis, trauma, valve repair/replacement, other cerebrovascular problems, other respiratory problems, and other diagnoses. 20 binary features were hence created to indicate the presence of each admission diagnosis, with ACS as the baseline, i.e., the comparison group. More details are available at the authors’ GitHub site^7^.

Another important feature that we defined was the APACHE Relative Confidence (ARC), which measures the distance from APACHE’s mortality prediction for each patient to a pre-defined cutoff, that is the cutoff determining whether or not a predicted probability is mapped to in-hospital death or survival. This distance was mapped to a range between 0 and 1, through dividing by the max possible distance, to reflect the relative confidence of APACHE in its mortality prediction probability. Formally, for a given patient *i*, let $\text {APACHE}_i$ denote the APACHE IVa’s predicted in-hospital mortality probability for patient *i*:

1 $$\begin{aligned} \text {ARC}_i = {\left\{ \begin{array}{ll} \frac{|\text {APACHE}_i - \text {cutoff}|}{\text {cutoff}} &{} \text {if APACHE}_i \le \text {cutoff} \\\\ \frac{\text {APACHE}_i - \text {cutoff}}{1-\text {cutoff}} &{} \text {if APACHE}_i > \text {cutoff} . \end{array}\right. } \end{aligned}$$

We hypothesized that a larger ARC (i.e., closer to 1) would suggest greater confidence in APACHE making a correct mortality prediction and thus lead to a smaller likelihood of both Type I and II errors. This speculation can also provide some justification why we may consider including ARC in our two types of APACHE error prediction models; that is, if the association is strong between the ARC and the APACHE error being predicted in a given model, then a quick check on ARC may suggest whether or not an APACHE error might have a reasonable chance of occurring (ARC near 0) or not occurring (ARC near 1) for a given patient. Alternatively, if there is greater interest in identifying a larger collection of original variables as being associated with an APACHE error, then one may consider excluding ARC in the models, assuming ARC has a strong association with errors occurring, as speculated above. That is, if ARC is not included, then perhaps some of the original predictor variables that are considered in the model (and either included in the APACHE score calculation or otherwise associated with APACHE score itself) may end up having explanatory value for the APACHE error occurrence and thus explicitly identified.

### Missing values

Missing values in our final cohort mainly came from those features related to lab tests, i.e. the tests which were not performed in the first 24 h.

If two or more measurements were observed for a certain lab test in the first 24 h, the worst value was chosen as what we defined above. If only one measurement was observed, we considered this value as the lab test’s worst value. If no measurement was observed, i.e. the lab test was not performed in the first 24 h, then this missingness was dealt with in one of the three following approaches: (1) replace the missing values with the pooled imputed values from multiple imputation, using 10 imputations; (2) replace the missing values with the median of the entire population and creating a binary flag for each lab test indicating if the test was not performed in the first 24 h^8^; (3) replace the missing values with grouped means based on a bucketing approach of the APACHE IVa scores^9^.

Specifically, in the third approach, the following steps were implemented to define the buckets:

1. Divided the patients into 10 equal-sized groups based on their APACHE IVa scores;
2. Replaced any missing lab test values in group *j* with the mean values in group *j*, $j = 1, 2, \ldots , 10$.

Previous studies have shown the superiority of multiple imputation (MI) in addressing missing values in different contexts, including patient registries^10^ and routine health data^11^, and thus we also utilized this method to address lab value missingness in our study. We also used the two easier-to-implement approaches mentioned above, i.e., fill missing values with population median or group means, respectively, as a basis of comparison.

Regarding missing APACHE values, we defer to the researchers who helped create the eICU database^5^, “Patients will not have an APACHE IV hospital mortality prediction if they satisfy exclusion criteria for APACHE IV (burns patients, in-hospital readmissions, some transplant patients), or if their diagnosis is not documented within the first day of their ICU stay”. Hence, these patients either were not intended to have a calculated APACHE score or were otherwise incapable of having it calculated without additional required information on their first 24-h diagnosis. Thus, our paper’s focus is only on those patients who were both eligible and able to have an APACHE score and associated in-hospital mortality prediction calculated, and, hence, we do not impute APACHE IVa for our analyses. This decision is also consistent with Cosgriff et al.^6^, discussed in the next subsection. Additionally, due to the proprietary nature of APACHE IVa model, our attempts to impute the APACHE IVa scores, as well as its subsequent in-hospital mortality predictions, were deemed invalid without knowing the specific model, including variable weights for the scores and link function for the predictions, that APACHE established between the two. Attempts at direct imputation of the missing APACHE in-hospital mortality prediction did not work either, producing very unstable results, likely due, to at least some extent, to lack of knowledge of the variable weights used in their prediction model.

### Cohort selection

We started by following the same cohort selection process as in the work by Cosgriff et al.^6^. Specifically, we removed the patient stays with no APACHE IVa scores or Glasgow coma scales, admitted due to lung or heart transplant or burns, age under 16 years, re-admissions or admitted from other ICU’s, and those without periodic vital records. This resulted in a remaining cohort of 134,946 stays, consisting of 115,350 unique patients.

Next, we imposed a further restriction to study only the last ICU stay in the final hospital stay for patients with multiple stays. However, the database did not always provide a way to allow a correct identification for the ordering of multiple hospital stays for one patient, primarily implemented due to privacy/confidentiality concerns. Consequently, we were not able to correctly identify the very last hospital stay of a subset of patients, and these patients’ records were subsequently removed from our analysis. In all, 97,547 confirmed unique final patient ICU stays remained.

As we were also interested in examining if a patient’s cure goal plan, physician intervention type and frequency, admission body mass index (BMI), or their medical histories would affect APACHE’s performance, patients without those records were removed. Of the 97,547 unique stays remaining, 89,322 had these required variables. See Fig. 1 for a flow chart summarizing how the final cohort was chosen.

20% of these 89,322 unique stays were randomly split off as a holdout final validation set. All initial model building, including model parameter tuning via (10-fold) cross validation, was conducted on the remaining 80%.

### Modeling approach

We started with the idea of using a generalized linear mixed-effects model with Lasso penalization^12^ to try to account for the heterogeneity from patients across different hospitals on the given APACHE prediction error (binary) response using a random intercept term. However, the heterogeneity exhibited through the estimated random effect variance was very small, and hence a random intercept term was not required. We then used a less complex generalized linear model, specifically logistic regression, with Lasso penalization. As is typically the case, the penalization was imposed in order to find a useful model that is more parsimonious than had no penalization been imposed. In addition, Lasso penalization, in general, leads to less biased final models than do stepwise regression techniques^13^.

For all models, the variables were standardized to mean 0 and variance 1 before modeling. An adaptive synthetic sampling (ADASYN) was utilized to avoid a prediction of all 0’s since there were extremely low positive rates for Type II errors^14^.

### Decisions

In our modeling approach, two parameters were required to be specified or tuned by the user: (1) the cutoff probability for mapping a dying versus surviving prediction; and (2) the penalization term (i.e., $\lambda$) in the Lasso model.

#### Cutoffs

When the APACHE prediction exceeds a certain pre-defined cutoff, we say the APACHE model is predicting the patient to die. We could have carefully tuned this cutoff with a great deal of computation, but we decided instead to examine the following cutoffs, with justification: 0.10, 0.50, and 0.33. We chose 0.10, as it is a cutoff sometimes used for defining patients at high risk^15^. 0.50 was an arbitrary choice, though a commonly used one, where we wished to examine how APACHE model performed when the cutoff was set to a high value, noting only about 5% of the patients were assigned a mortality prediction greater than 0.50. Finally, 0.33 was the calibrated value which made the percentage of patients with APACHE mortality predictions greater than 0.33 (9.92%) coincide with the observed mortality rate in the cohort (9.91%).

#### Penalization in Lasso regression

With the goal to select the most significant features which may indicate an APACHE error, we chose a penalization value that would result in the most parsimonious model while not making a major compromise in model performance. There are two commonly used approaches in the literature for choosing the penalization term: one, choose the largest penalization term within one standard error from the one that produced the best evaluation metric (e.g.,^16,17^); or two, choose the largest penalization term within the 95% confidence interval of the one that produced the best evaluation metric. The penalization term chosen in approach two is always larger than the one chosen using approach one and thus always produces a more parsimonious model (e.g.,^18^).

We implemented the initial $\lambda$ selection within each type of error prediction and on the training data using each of the three missing handling approaches, where we tuned the choice of $\lambda$ using 10-fold cross validation within the 80% model selection sample. This process was repeated for five replications, and the evaluation metrics on the holdout cohort as well as the variables selected were found consistent throughout the five replications. The model performance was evaluated on the 20% holdout set for the first replication with summary performance metrics displayed in Table 1 of the “Results” section. Other replications’ performance metrics are available in Supplementary Information.

A summary of all our modeling steps can also be found in Supplementary Information.

### Ethics approval and consent to participate

Both authors were required to complete the CITI “Data or Specimens Only Research” online course, and complete a subsequent application, in order to be approved to gain access to the de-identified eICU database used for the secondary data analyses in this paper.

### Consent for publication

No consent was required, due to only de-identified records being available in the eICU database.

## Results

### ARC

**Figure 3:**
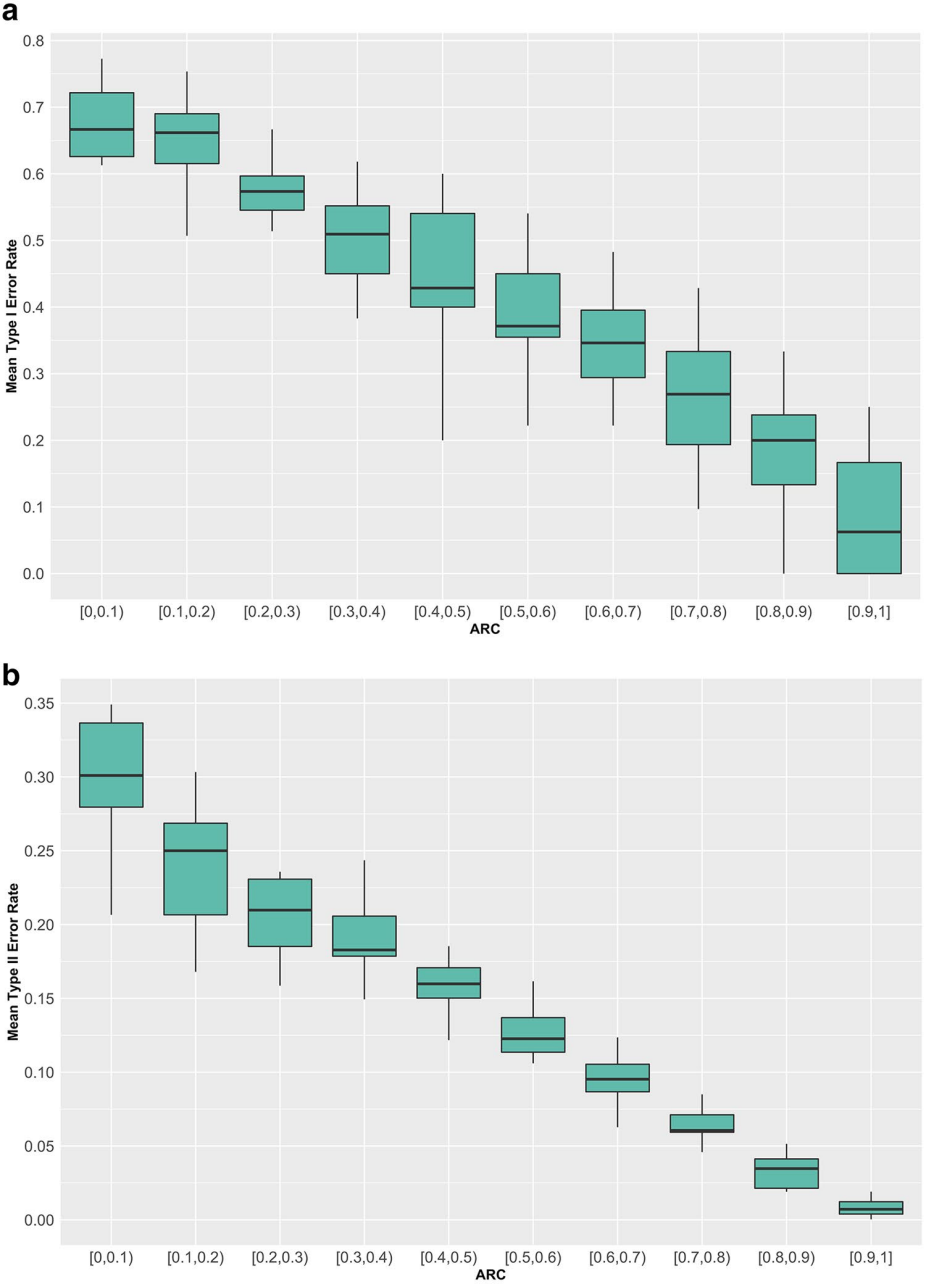
(**a**) Type I error rate against different ARC values. (**b**) Type II error rate against different ARC values.

Figure 3a,b displays the Type I/II error rate against ARC values. Here, we included the example using the APACHE in-hospital mortality prediction probability cutoff equal to 0.33, noting the importance of this cutoff mentioned in “Methods” in terms of calibration of observed mortality rate of our cohort, not to mention that the result patterns with the other two cutoffs are similar.

It is clear to observe from Fig. 3a,b a monotonic decreasing error rate for both Type I and Type II Errors, as ARC increases from 0 to 1, which aligns with our a priori belief.

### Performance metrics

Tables 1, 2 and 3 summarize the performance metrics and features selected for the two types of errors on training sets obtained by using 0.33 as the cutoff value, from each of the three missing handling approach. We only present here the results from the first replication, as the results from other replications are very consistent. Unsurprisingly, some of the performance metrics change as a result of a change of cutoff. All these additional results can be found in Supplementary Information.

**Table 1:** Performance metrics for Type I/II errors using cutoff 0.33.

Missing handling	Error	Pop. ErrorRate$^{1}$ (%)	AUROC$^{2}$	AUPRC$^{3}$	Precision	Recall	F-score$^{4}$	Accuracy$^{5}$
Multiple imputation	Type I	51.56	0.7030	0.6802	0.6248	0.7754	0.6920	0.6426
	Type II	5.66	0.8195	0.2185	0.2436	0.2705	0.2564	0.9109
Fill with median	Type I	51.56	0.6996	0.6759	0.6246	0.7655	0.6879	0.6403
	Type II	5.66	0.8172	0.2045	0.1655	0.6583	0.2645	0.7922
Fill with group mean	Type I	51.56	0.6934	0.6732	0.6242	0.7699	0.6895	0.6409
	Type II	5.66	0.8197	0.2082	0.1655	0.6583	0.2645	0.7922

$^{1}$Pop. ErrorRate is the true observed APACHE error rate in the entire population.

$^{2}$AUROC is the area under the receiver operating characteristic curve.

$^{3}$AUPRC is the area under the precision–recall curve.

$$^{4}$$ $$\text {F-score}=2\times \frac{\text {Precision} \times \text {Recall}}{\text {Precision} + \text {Recall}}$$

$^{5}$Accuracy $= \frac{TP+TN}{TP+TN+FP+FN}$, where $TP=$ true positive, $TN=$ true negative, *FP* = false positive, and $FN =$ false negative.

The results in Table 1 were calculated on the randomly selected 20% holdout validation sample, following the cross-validation step to produce the Lasso regularization parameter $\lambda$ and the best model using internal validation on the other 80%, as described in “Methods”. Specifically, in Table 1, we observe that the APACHE’s Type II error rate (i.e., 5.66%) was extremely low in our cohort, leading to a high AUROC, as simply predicting everything to 0 would have resulted in an over 94% AUROC. In such a scenario where we have a very imbalanced target variable, it is advised to examine the AUPRC or F-score instead of the AUROC or the crude accuracy since both AUPRC and F-score incorporate both recall and precision into evaluating the model performance^19^. The AUPRC and F-score for the model predicting Type II errors were much lower than that from predicting Type I errors, suggesting more difficulties in predicting APACHE’s failure when patients received lower APACHE scores following the first 24 h of their ICU stay. This deficiency in the prediction’s precision, recall, AUPRC and F-score could be also attributable to the fact that we had very low prevalence rate of Type II errors (only 5.66%), making it much more vulnerable to a single false prediction than a model with higher prevalence error rate (e.g., the other model predicting Type I errors).

**Table 2:** Features selected for Type I error when using cutoff 0.33.

Missing handling	Features—Type I error	Coefficients$^{1}$	Coefficients$^{2}$	SE$^{2}$	Z-ratio$^{2}$	95% CI$^{2}$
Multiple imputation	ARC	− 0.2282	− 0.5467	0.029	19.2	(− 0.603, − 0.491)
	Mean SpO2	0.0434	0.6442	0.047	13.8	(0.552, 0.736)
	Worst (max) lactate	− 0.1583	− 0.4383	0.033	13.2	(− 0.503, − 0.373)
Fill with median	ARC	− 0.3022	− 0.6033	0.028	21.3	(− 0.659, − 0.548)
	Mean SpO2	0.0689	0.6167	0.045	13.6	(0.528, 0.705)
	Worst (Max) Lactate	− 0.1211	− 0.4097	0.033	12.4	(− 0.475, − 0.345)
Fill with group mean	ARC	− 0.2573	− 0.5808	0.029	20.3	(− 0.637, − 0.525)
	Mean SpO2	0.0290	0.6215	0.045	13.7	(0.533, 0.710)
	Worst (Max) Lactate	-0.2034	-0.3949	0.033	12.1	(− 0.459, − 0.331)

$^{1}$Coefficients calculated from fitting the Lasso feature selection model.

$^{2}$Coefficients, SE, Z-ratios, and the 95% CIs calculated by ordinary logistic regression using the selected features from Lasso; features sorted by Z-ratios, where Z-ratio $= |\text {Coefficient}/SE|$.

**Table 3:** Features selected for Type II error when using cutoff 0.33.

Missing handling	Features—type II error	Coefficients$^{1}$	Coefficients$^{2}$	SE$^{2}$	Z-ratio$^{2}$	95% CI$^{2}$
Multiple imputation	ARC	− 0.7521	− 1.1789	0.009	133.6	(− 1.196, − 1.162)
	Worst (Min) albumin	− 0.1349	− 0.4775	0.008	61.1	(− 0.493, − 0.462)
	Mean heart rate	0.0223	0.3907	0.007	52.6	(0.376, 0.405)
Fill with median	ARC	− 0.7378	− 1.2424	0.009	143.5	(− 1.259, − 1.225)
	Worst (Min) albumin	− 0.0444	− 0.4332	0.008	56.5	(− 0.448, − 0.418)
Fill with group mean	ARC	− 0.6891	− 1.1704	0.009	135.5	(− 1.187, − 1.153)
	Worst (Min) albumin	− 0.0667	− 0.4388	0.008	57.0	(− 0.454, − 0.424)

$^{1}$Coefficients calculated from fitting the Lasso feature selection model.

$^{2}$Coefficients, SE, Z-ratios, and the 95% CIs calculated by ordinary logistic regression using the selected features from Lasso; Features sorted by Z-ratios, where Z-ratio $= |\text {Coefficient}/SE|$.

In Table 2, we can see only a few predictors remain after implementing the Lasso penalization for predicting Type I error, i.e., the error observed when APACHE predicts a death before discharge. Unsurprisingly, ARC is highly significant with a negative coefficient, as the larger the ARC, the less likely a Type I error is bound to occur. Similarly, the lower the max lactate, and the higher the mean oxygen saturation (SpO2), the higher the chance of APACHE making a Type I error.

In Table 3, there are only two additional predictors beyond ARC that made the final model for predicting Type II error, i.e., the error observed when APACHE does not predict a death before discharge, in the model where missing lab values were handled by multiple imputation. Like for the model predicting Type I error, ARC ends up being the strongest variable, with higher ARC being associated with a lower likelihood of a Type II error occurring. Other strong variables include worst (minimum) albumin rate with a negative coefficient, and mean heart rate with a positive coefficient; in other words, lower worst albumin and higher mean heart rate are both associated with a greater chance of someone dying when APACHE initially predicts in-hospital survival.

Tables 4, 5 and 6 summarize the performance metrics and features that result if ARC is not included in the Lasso model. These are the analogs of Tables 1, 2 and 3, respectively, where ARC was included in the modeling. Note the extra variables included in the final models when APACHE Relative Confidence is not considered. We address this point again in “Discussion”.

**Table 4:** Performance metrics for Type I/II errors using cutoff 0.33, with ARC removed.

Missing handling	Error	Pop. ErrorRate$^{1}$ (%)	AUROC$^{2}$	AUPRC$^{3}$	Precision	Recall	F-score$^{4}$	Accuracy$^{5}$
Multiple imputation	Type I	51.56	0.6873	0.6530	0.6064	0.8637	0.7126	0.6392
	Type II	5.66	0.7888	0.2123	0.1400	0.7097	0.2339	0.7360
Fill with median	Type I	51.56	0.6945	0.6642	0.6041	0.8637	0.7110	0.6364
	Type II	5.66	0.7935	0.2120	0.1408	0.7360	0.2364	0.7300
Fill with group mean	Type I	51.56	0.6973	0.6647	0.6065	0.8506	0.7081	0.6369
	Type II	5.66	0.7929	0.2060	0.1339	0.7360	0.2266	0.7149

$^{1}$Pop. ErrorRate is the true observed APACHE error rate in the entire population.

$^{2}$AUROC is the area Under the receiver operating characteristic curve.

$^{3}$AUPRC is the area Under the precision–recall curve.

$$^{4}$$ $$\text {F-score}=2\times \frac{\text {Precision} \times \text {Recall}}{\text {Precision} + \text {Recall}}$$.

$^{5}$Accuracy $= \frac{TP+TN}{TP+TN+FP+FN}$, where $TP=$ true positive, $TN=$ true negative, *FP* = false positive, and *FN* = false negative.

**Table 5:** Features selected for Type I error when using cutoff 0.33, with ARC removed.

Missing handling	Features—Type I Error	Coefficients$^{1}$	Coefficients$^{2}$	SE$^{2}$	Z-ratio$^{2}$	95% CI$^{2}$
Multiple imputation	Worst (Max) Lactate	− 0.3040	− 0.5063	0.036	13.9	(− 0.577, − 0.435)
	Mean SpO2	0.1452	0.5418	0.045	12.2	(0.454, 0.629)
	Care limitation$^{3}$	− 0.0367	− 0.3095	0.031	10.1	(− 0.370, − 0.249)
	Worst (Min) pH	0.0210	0.1641	0.031	5.2	(0.102, 0.226)
Fill with median	Mean SpO2	0.1399	0.5563	0.044	12.5	(0.469, 0.643)
	Worst (Max) Lactate	− 0.2325	− 0.4027	0.035	11.5	(− 0.471, − 0.334)
	Care limitation$^{3}$	− 0.0382	− 0.3315	0.031	10.5	(− 0.393, − 0.270)
	Cardiac arrest at admission	− 0.0187	− 0.2533	0.027	9.4	(− 0.306, − 0.201)
	Vasopressor$^{4}$	− 0.0174	− 0.1914	0.027	7.1	(− 0.244, − 0.139)
	Worst (Min) pH	0.0195	0.1003	0.031	3.2	(0.04, 0.161)
Fill with group mean	Mean SpO2	0.1325	0.5513	0.045	12.4	(0.464, 0.639)
	Worst (Max) Lactate	− 0.2565	− 0.4195	0.035	12.0	(− 0.488, − 0.351)
	Care limitation$^{3}$	− 0.0369	− 0.3324	0.032	10.5	(− 0.394, − 0.271)
	Cardiac arrest at admission	− 0.0089	− 0.2415	0.027	9.0	(− 0.294, − 0.189)
	Vasopressor$^{4}$	− 0.0287	− 0.1989	0.027	7.3	(− 0.252, − 0.146)
	Worst (Min) pH	0.0231	0.1007	0.031	3.2	(0.039, 0.162)

$^{1}$Coefficients calculated from fitting the Lasso feature selection model.

$^{2}$Coefficients, SE, Z-ratios, and the 95% CIs calculated by ordinary logistic regression using the selected features from Lasso; Features sorted by Z-ratios, where Z-ratio $= |\text {Coefficient}/SE|$.

$^{3}$Care Limitation is how often the care limitation(s) recorded in the care planning was required to change for the patient.

$^{4}$Whether vasopressor was provided to the patient within the first 24 h.

**Table 6:** Features selected for Type II error when using cutoff 0.33, with ARC removed.

Missing handling	Features—Type II error	Coefficients$^{1}$	Coefficients$^{2}$	SE$^{2}$	Z-ratio$^{2}$	95% CI$^{2}$
Multiple imputation	Age	0.1210	0.6270	0.008	76.4	(0.611, 0.643)
	Ventilated in day 1	0.0345	0.4702	0.007	66.5	(0.456, 0.484)
	Worst (Min) albumin	− 0.2785	− 0.4665	0.008	57.8	(− 0.482, − 0.451)
	Mean respiratory rate	0.0712	0.3867	0.008	50.1	(0.372, 0.402)
	Worst (Max) lactate	0.0455	0.4366	0.010	45.2	(0.418, 0.456)
	Mean heart rate	0.0307	0.3481	0.008	43.2	(0.332, 0.364)
	Worst (Max) BUN$^{4}$	0.0602	0.2983	0.008	37.8	(0.283, 0.314)
	Mean aperiodic vital	− 0.0143	− 0.2443	0.008	32.1	(− 0.259, -0.229)
Fill with median	Age	0.1346	0.6137	0.008	77.5	(0.598, 0.629)
	Ventilated in Day 1	0.0342	0.4700	0.007	67.6	(0.456, 0.484)
	Worst (Min) albumin	− 0.1891	− 0.4120	0.008	51.8	(− 0.428, − 0.396)
	Mean heart rate	0.0428	0.3760	0.008	48.2	(0.361, 0.391)
	Mean respiratory rate	0.0423	0.3452	0.008	46.0	(0.330, 0.360)
	Mean aperiodic vital	− 0.0545	− 0.2912	0.007	39.3	(− 0.306, − 0.277)
	Worst (Max) BUN$^{4}$	0.0138	0.2465	0.008	32.1	(0.231, 0.262)
	Whether Lactate test provided$^{5}$	− 0.0472	− 0.1944	0.007	26.5	(− 0.209, − 0.18)
Fill with group mean	Age	0.1344	0.5748	0.008	73.0	(0.559, 0.590)
	Ventilated in Day 1	0.0368	0.4591	0.007	66.5	(0.446, 0.473)
	Worst (Min) albumin	− 0.2685	− 0.4688	0.008	59.2	(− 0.484, − 0.453)
	Mean heart rate	0.0440	0.3882	0.008	50.1	(0.373, 0.403)
	Mean respiratory rate	0.0594	0.3667	0.008	48.8	(0.352, 0.381)
	Mean aperiodic vital	− 0.0546	− 0.3007	0.007	40.5	(− 0.315, − 0.286)
	Worst (Max) BUN$^{4}$	0.0318	0.2819	0.008	36.6	(0.267, 0.297)

$^{1}$Coefficients calculated from fitting the Lasso feature selection model.

$^{2}$Coefficients, SE, Z-ratios, and the 95% CIs calculated by ordinary logistic regression using the selected features from Lasso; Features sorted by Z-ratios, where Z-ratio $= |\text {Coefficient}/SE|$.

$^{4}$BUN: Blood Urea Nitrogen test.

$^{5}$ Whether a lactate test was provided within the first 24 h.

Comparing Tables 1 to 4, Tables 2 to 5, and Tables 3 to 6, respectively, we observed that whether or not ARC was included in the model had very small impact on the overall model performance, in terms of F1-scores. Specifically, adding ARC has slightly improved the F1-scores in models predicting Type II errors but slightly decreased F1-scores in the ones predicting Type I errors. However, including ARC significantly increased the parsimony of the model, meaning that fewer features were needed to maintain the same (or even slightly higher) level of predicting power of the model, especially in the prediction of Type II errors. In addition, as displayed in the final column of Tables 1 and 3, the accuracy of the predictions was improved when ARC was included, though only marginally so for predicting Type I errors.

Given the difference in the predictors obtained in the models with or without ARC, we refer to the predictors that made the final model in the presence of ARC as the ***primary*** set of predictors, as they were strong enough to compete with ARC and were also the ones with strongest predicting power in the models without ARC. We then refer to the other predictors that made only the models without ARC as the ***secondary*** set of predictors.

### Sensitivity to missing data approach chosen

Among all the lab tests and all patients, 1,196,502 (39.4%) out of the 3,036,948 patient$\times$lab combinations are missing (i.e., lab test not performed in the first 24 h); however, the missing percentages range from 3 (Creatinine) to 95% (Amylase). Despite this huge range of missingness, we observe fairly consistent results on the models obtained from the three distinct missing handling approaches we implemented.

Where there is at least one measurement for a patient$\times$lab combination within the first 24 h in the ICU, 613,711 (20.2%) patient$\times$lab combinations have exactly one measurement, and 1,226,735 (40.4%) have two or more measurements.

## Discussion

The APACHE scoring approach (specifically, now, APACHE IVa) is considered to be a good prediction tool in intensive care units for speculating on outcomes such as in-hospital mortality and length of stay based on data collected within the first 24 h of a patient’s stay^1^. Our goal in this paper was to use a large heterogeneous multi-hospital ICU study^5^ to attempt to identify some predictors of patient information collected in the first 24 h that might be predictive of when the APACHE prediction may go wrong regarding its speculation on in-hospital mortality.

As there are two ways of APACHE leading to prediction errors, with these predictions likely leading to different approaches to patient care, we thought it was important to separate the two errors as: (i) Type I errors, or false positives (i.e., APACHE predicting in-hospital mortality, but the patient is discharged alive), and (ii) Type II error, or false negatives (i.e., APACHE predicting being discharged alive, but the patient dies at some point during their hospital stay).

We used an out-of-sample validation approach, following a model-building step using cross-validation, to model these two errors separately with multiple logistic regression with Lasso penalization. The differential results, seen in Tables 1, 2 and 3 (as well as in Tables 4, 5 and 6) further support our approach to model the two APACHE prediction errors separately.

Some of the results in this study are unsurprising, such as seeing the so-called *ARC* variable, which represents the absolute relative difference between the APACHE prediction for a given patient and a pre-determined cutoff score used to classify a patient into either an in-hospital death or survival prediction, being very important for both types of errors. Specifically, the higher the ARC, the less likely APACHE will make an error. This suggests having intensivists pay close attention to lower ARC scores, as these are the ones that appear to be more subject to both types of errors (see, again, Fig. 3a,b). We are not suggesting that such checking of ARC should replace any established clinical practice, but that it could potentially help supplement practice already in place.

No single variable, aside from ARC, made both final models, among the models where ARC was included as a predictor. For Type I error, only worst (max) lactate and mean SpO2 made the final model, whereas, for Type II error, only worst (min) albumin and mean heart rate made the final model as our primary set of indicators. For a given APACHE prediction, according to our findings, the small number of predictors listed in either Tables 2 or 3 should be given added scrutiny in the ICU. For example, related to Type II error, if a patient has low minimum albumin and receives a sufficiently low APACHE score to suggest that the patient will survive the current hospital stay, perhaps greater attention should be given to that patient than might otherwise be provided given the low (APACHE) score.

We also presented results from models that did not include ARC as a predictor. This allows us to compare the performance of models, as well as parameter estimates, when including or excluding ARC. Unsurprisingly, the results for the non-ARC models result in a larger number of predictors. Notwithstanding any differences in predictive performance for seeing the success (or lack thereof) of APACHE’s prediction for a given patient, the choice of ARC’s inclusion may be dependent on a given researcher’s preference: if ARC is excluded, i.e., the extremeness of the APACHE (IVa) prediction of in-hospital mortality in model building is ignored, we may uncover additional variables of interest in the prediction of APACHE making incorrect classifications, but when ARC is included, the result will be a more parsimonious final model that still has relatively good predictive performance for identifying when APACHE may make a prediction error for in-hospital mortality.

Specifically, in the models predicting Type I errors but with ARC removed (Table 5), we observed that more changes in the nurses and physicians’ care limitation notes and worse pH in the first 24 h can be also indicative of a potential failure of APACHE, provided that the predicted mortality was relatively high. More secondary predictors were selected when ARC was removed in the prediction of Type II errors (Table 6). Interestingly, in this set of models, the patient’s age and whether or not the patient was ventilated surpassed the primary set of predictors (i.e., worst albumin and mean heart rate) and produced the largest coefficients among all predictors. These two variables were also used in the calculation of an APACHE IVa score as well as its in-hospital mortality prediction, so their importance may be taken away by ARC in the parallel models. In addition to these two most important predictors, we also observed higher respiratory rate, worse lactate, worse BUN, and lower mean aperiodic vitals to increase the chance of APACHE producing a Type II error.

We should mention some limitations of this study and areas of future work. In terms of limitations, though we utilized a large multi-hospital cohort from over 200 ICU’s, this was still a U.S.-based cohort, and the results may not necessarily replicate in other countries. It will be interesting to see true external validation^20^ of the models fit here in ICU’s in other countries, noting that evaluating performance of our models on the holdout validation sample amounts to a pseudo-external validation approach, and actually, within eICU, a form of external validation could be done by holding out randomly selected hospitals, as opposed to patients. This investigation can be an area of future work.

We also had to make a number of decisions in our cohort selection and modeling efforts, as described in “Methods”, some of which could be potentially scrutinized further (e.g., our decision on a cutoff value) to ensure the robustness of the results presented here. Also, it is well known that missing data are a problem in modeling electronic health records^21^. The three distinct missing data approaches we used led to similar results, but this does not guarantee a modification of one of the current approaches (e.g., changing the bin size of the second approach we used^9^) or a different missing data approach might not lead to more accurate results for these data. This could be a further area of research in dealing with the prediction of when APACHE may lead to errors. Simulation studies could be helpful for such an investigation.

In this paper, we wanted to directly investigate variables associated with APACHE’s occasional failure for predicting in-hospital mortality. However, some may prefer an approach that directly models the prediction of in-hospital mortality, while adjusting for APACHE score, and see variables that remain important in the model after controlling for APACHE. Such differences in approach could be compared in future work, though the separation of the two types of prediction errors discussed in this paper may be better done by modeling such errors separately. The key issue for this latter approach is then determinining the appropriate APACHE prediction probability cutoff value (for determining if APACHE is deciding death or survival), and this cutoff choice might need to be part of a tuning process for other datasets.

Finally, we should reiterate that we only focused upon information collected in the first 24 h of an ICU’s patient stay, based on the information that is used in generating APACHE scores. But an additional area of future work is to identify what happened to the patient after the first 24 h that tends to be associated with the original APACHE prediction leading to an error. This could potentially be done in a causal inference framework^22^. This said, it is still important to know what variables are observed early that might lead to an APACHE prediction error, especially in the case where a low APACHE score might suggest less scrutiny of such patients is required as compared to those with higher scores. Hopefully, the work presented here is informative on its own, and that it will also lead to further research into the use, inspection, and potential improvement, of APACHE predictions of in-hospital mortality of ICU patients.

## Conclusions

In conclusion, we have determined the variables available within the first 24 h of a patient’s ICU stay that may be indicative of the APACHE IVa scoring system’s occasional prediction errors, through logistic regression modeling with Lasso penalization. The variable, *ARC*, was found to be the most important predictor, with higher (closer to 1) ARC being associated with a lower likelihood of both types of APACHE prediction errors occurring. ARC could prove to be an efficient check on when APACHE prediction errors on in-hospital mortality may occur. Our analyses also suggested that a lower max lactate and higher mean oxygen saturation (SpO2) are associated with a higher chance of APACHE making a Type I error; and that lower worst albumin and higher mean heart rate are associated with a greater chance of a Type II error. However, we also generated models that did not include ARC, in order that potentially a longer list of variables could be identified as being associated with either type of APACHE prediction error; this indeed proved to be the case, including age and ventilation for Type II error, and care limitation for Type I error.

## Supplementary Information

Supplementary Information.

## Data Availability

The datasets generated and/or analysed during the current study are available in the eICU Collaborative Research Database. Researchers seeking to use this database are required to apply, with details at the following website: https://eicu-crd.mit.edu/gettingstarted/access/.

## Abbreviations

ACS: Acute coronary syndrome
ADASYN: Adaptive synthetic sampling
APACHE: Acute physiology and chronic health evaluation
ARC: APACHE relative confidence
ARF: Acute renal failure
AUPRC: Area under the precision–recall curve
AUROC: Area under the receiver operating characteristic curve
BMI: Body mass index
BUN: Blood urea nitrogen
CABG: Coronary artery bypass grafting
CHF: Congestive heart failure
CI: Confidence interval
CVA: Cerebrovascular accident/stroke
DKA: Diabetic ketoacidosis
GI: Gastrointestinal
ICU: intensive care unit
Lasso: Least absolute shrinkage and selection operator
PNA: Pneumonia
SE: Standard error
