# Supplementary material for: Identifying early-measured variables associated with APACHE IVa providing incorrect in-hospital mortality predictions for critical care patients
Source: Sci Rep. 2021 Nov 12;11:22203. doi: 10.1038/s41598-021-01290-7 (PMC8589984; doi:10.1038/s41598-021-01290-7)
Supplement: Supplementary file 1 — Supplementary Information. [file 41598_2021_1290_MOESM1_ESM.pdf]

Identifying early-measured variables associated with APACHE IVa providing incorrect in-hospital mortality predictions for critical care patients

Shuo Feng[1] **and Joel A. Dubin**[1, 2, *]

[1]Department of Statistics and Actuarial Science, University of Waterloo, Waterloo, ON, Canada
[2]School of Public Health Sciences, University of Waterloo, Waterloo, ON, Canada
[*]jdubin@uwaterloo.ca

Supplementary Information

S.1 Summarization of modeling steps

(A) As conveyed in Figure 1, once the full cohort (of 89,322 unique patient ICU stays) was identified, we randomly split the model into an 80% group for Lasso logistic regression model building, and the remaining 20% were held out to be used as an out-of-sample validation set, for the purpose of evaluating the final model (one model for each of the two responses of interest).

(B) From the 80% model building dataset, missing values were imputed and replaced by each of three methods discussed. We then prepared the completed data for 10-fold cross-validation, i.e., creating folds $F_1, ..., F_{10}$, for the purposes of tuning the Lasso penalty parameter λ.

(C) For a given cutoff, either 0.10, 0.33, or 0.50, we then used the *cv.glmnet* function from package *glmnet*[1], version 4.0-2, in R[2], version 4.0.2, which automatically picks a sequence of λ values that minimized deviance loss in each fold F_k, $k = 1, ..., 10$, then averages over each fold to find the optimal λ.

(D) The approach found in step (C) is often associated with a small amount of regularization (i.e., keeping in many variables). Hence, our λ was chosen in one of the two approaches (1) the λ that results in the most parsimonious model within 1 standard deviation from the best λ from *cv.glmnet*; or (2) the λ that results in the most parsimonious model within a 95% confidence interval from the best λ from *cv.glmnet*. We did this separately for each of the two responses of interest.

(E) For a given response, either a Type I or a Type II error, using its associated λ chosen from (D), we ran a Lasso logistic regression model on the full 80% model building sample. Once we identified the predictors that were not screened out, we re-ran the model with those predictors only in a regular logistic regression to produce the results, including estimated regression coefficients, standard errors, and 95% confidence intervals, in Tables 2 and 3, respectively, for cutoff 0.33 of Type I or Type II errors, and for each missing handling approach. The performance metrics were obtained by testing the Lasso model on the 20% hold-out validation set. Their results were summarized in Table 1.

(F) Next, we replicated step (E) above but with the removal of ARC in the model fitting, with results shown in Tables 4, 5, and 6.

(G) The results displayed in Tables 1 through 3 from step (E) are for a single replication, but we did run five replications in total to check for consistency of these results, which can be found below. The results for other cutoffs, i.e., cutoff 0.10 and 0.50 are also available in below.

S.2 Other Replications

Performance Metrics

Table S.2.1. Performance metrics for Type I/II errors using cutoff 0.10 of all five replications

Error	Pop. ErrorRate[1]	Missing Handling	Replication	AUROC[2]	AUPRC[3]	Precision	Recall	F-score[4]	Accuracy[5]
Type I	74.34%	Multiple Imputation	1	0.7498	0.8765	0.7792	0.9806	0.8684	0.7786
			2	0.7452	0.8735	0.7696	0.9881	0.8652	0.7708
			3	0.7454	0.8736	0.7693	0.9881	0.8650	0.7704
			4	0.7447	0.8735	0.7674	0.9895	0.8644	0.7689
			5	0.7445	0.8735	0.7647	0.9927	0.8639	0.7671
		Fill with Median	1	0.7515	0.8791	0.7805	0.9782	0.8683	0.7790
			2	0.7469	0.8769	0.7770	0.9801	0.8668	0.7757
			3	0.7469	0.8769	0.7770	0.9801	0.8668	0.7757
			4	0.7469	0.8769	0.7770	0.9801	0.8668	0.7757
			5	0.7469	0.8769	0.7770	0.9801	0.8668	0.7757
		Fill with Group Mean	1	0.7510	0.8787	0.7810	0.9785	0.8687	0.7797
			2	0.7466	0.8762	0.7772	0.9806	0.8671	0.7762
			3	0.7466	0.8762	0.7772	0.9806	0.8671	0.7762
			4	0.7466	0.8762	0.7772	0.9806	0.8671	0.7762
			5	0.7466	0.8762	0.7772	0.9806	0.8671	0.7762
Type II	2.42%	Multiple Imputation	1	0.7475	0.0618	0.0947	0.0954	0.0951	0.9575
			2	0.7471	0.0614	0.0986	0.0989	0.0988	0.9578
			3	0.7459	0.0596	0.0912	0.0919	0.0915	0.9574
			4	0.7465	0.0627	0.0954	0.0954	0.0954	0.9577
			5	0.7471	0.0616	0.0951	0.0954	0.0952	0.9576
		Fill with Median	1	0.7680	0.0789	0.0549	0.6855	0.1017	0.7167
			2	0.7698	0.0804	0.0551	0.6855	0.1021	0.7180
			3	0.7701	0.0806	0.0557	0.6926	0.1032	0.7184
			4	0.7739	0.0829	0.0554	0.6855	0.1025	0.7192
			5	0.7703	0.0807	0.0554	0.6890	0.1026	0.7180
		Fill with Group Mean	1	0.7683	0.0796	0.0554	0.6926	0.1026	0.7168
			2	0.7726	0.0829	0.0558	0.6926	0.1033	0.7187
			3	0.7695	0.0801	0.0558	0.6926	0.1032	0.7187
			4	0.7728	0.0832	0.0559	0.6926	0.1034	0.7191
			5	0.7717	0.0821	0.0553	0.6855	0.1024	0.7191

[1] Pop. ErrorRate is the true observed APACHE error rate in the entire population.
[2] AUROC is the Area Under the Receiver Operating Characteristic curve.
[3] AUPRC is the Area Under the Precision-Recall Curve.
[4] F-score $= 2 \times \frac{\text{Precision} \times \text{Recall}}{\text{Precision} + \text{Recall}}$
[5] Accuracy $= \frac{TP+TN}{TP+TN+FP+FN}$, where TP = true positive, TN = true negative, FP = false positive, and FN = false negative.

Table S.2.2. Performance metrics for Type I/II errors using cutoff 0.33 of all five replications

Error	Pop. ErrorRate[1]	Missing Handling	Replication	AUROC[2]	AUPRC[3]	Precision	Recall	F-score[4]	Accuracy[5]
Type I	51.56%	Multiple Imputation	1	0.7030	0.6802	0.6248	0.7754	0.6920	0.6426
			2	0.7031	0.6803	0.6259	0.7754	0.6926	0.6437
			3	0.7028	0.6801	0.6242	0.7754	0.6916	0.6420
			4	0.7009	0.6780	0.6231	0.7754	0.6910	0.6409
			5	0.6959	0.6726	0.6208	0.7623	0.6843	0.6358
		Fill with Median	1	0.6996	0.6759	0.6246	0.7655	0.6879	0.6403
			2	0.6947	0.6719	0.6241	0.7677	0.6885	0.6403
			3	0.6973	0.6740	0.6217	0.7634	0.6853	0.6369
			4	0.6996	0.6759	0.6246	0.7655	0.6879	0.6403
			5	0.6947	0.6719	0.6241	0.7677	0.6885	0.6403
		Fill with Group Mean	1	0.6996	0.6759	0.6246	0.7655	0.6879	0.6403
			2	0.6988	0.6769	0.6262	0.7710	0.6911	0.6431
			3	0.6988	0.6769	0.6262	0.7710	0.6911	0.6431
			4	0.6988	0.6769	0.6262	0.7710	0.6911	0.6431
			5	0.6964	0.6756	0.6253	0.7699	0.6901	0.6420
Type II	5.66%	Multiple Imputation	1	0.8195	0.2085	0.2436	0.2705	0.2564	0.9109
			2	0.8138	0.1975	0.2321	0.2519	0.2416	0.9102
			3	0.8117	0.1941	0.2327	0.2508	0.2414	0.9105
			4	0.8145	0.1999	0.2366	0.2563	0.2461	0.9108
			5	0.8117	0.1941	0.2363	0.2464	0.2413	0.9120
		Fill with Median	1	0.8172	0.2045	0.1655	0.6583	0.2645	0.7922
			2	0.8226	0.2163	0.1669	0.6616	0.2666	0.7934
			3	0.8225	0.2160	0.1674	0.6637	0.2673	0.7935
			4	0.8171	0.2043	0.1654	0.6583	0.2644	0.7921
			5	0.8227	0.2164	0.1664	0.6616	0.2660	0.7927
		Fill with Group Mean	1	0.8197	0.2082	0.1655	0.6583	0.2645	0.7922
			2	0.8117	0.1941	0.1768	0.5904	0.2722	0.8208
			3	0.8205	0.2100	0.1658	0.6583	0.2649	0.7926
			4	0.8206	0.2101	0.1656	0.6594	0.2647	0.7921
			5	0.8117	0.1941	0.1771	0.5904	0.2725	0.8211

[1] Pop. ErrorRate is the true observed APACHE error rate in the entire population.
[2] AUROC is the Area Under the Receiver Operating Characteristic curve.
[3] AUPRC is the Area Under the Precision-Recall Curve.
[4] F-score $= 2 \times \frac{\text{Precision} \times \text{Recall}}{\text{Precision} + \text{Recall}}$
[5] Accuracy $= \frac{TP+TN}{TP+TN+FP+FN}$, where $TP =$ true positive, $TN =$ true negative, FP = false positive, and FN = false negative.

Table S.2.3. Performance metrics for Type I/II errors using cutoff 0.50 of all five replications

Error	Pop. ErrorRate[1]	Missing Handling	Replication	AUROC[2]	AUPRC[3]	Precision	Recall	F-score[4]	Accuracy[5]
Type I	39.98%	Multiple Imputation	1	0.6980	0.5723	0.5064	0.7394	0.6011	0.6112
			2	0.6992	0.5750	0.5113	0.7207	0.5982	0.6164
			3	0.7054	0.5830	0.5043	0.7846	0.6139	0.6091
			4	0.7022	0.5793	0.5091	0.7447	0.6048	0.6143
			5	0.7046	0.5823	0.5054	0.7447	0.6022	0.6101
		Fill with Median	1	0.7054	0.5768	0.5213	0.7154	0.6031	0.6270
			2	0.6911	0.5568	0.5185	0.7074	0.5984	0.6238
			3	0.7023	0.5726	0.5253	0.7181	0.6067	0.6312
			4	0.6976	0.5678	0.5233	0.7154	0.6045	0.6291
			5	0.6946	0.5612	0.5214	0.7128	0.6022	0.6270
		Fill with Group Mean	1	0.7140	0.5831	0.5290	0.7527	0.6213	0.6365
			2	0.7113	0.5798	0.5219	0.7287	0.6082	0.6280
			3	0.7111	0.5793	0.5208	0.7340	0.6093	0.6270
			4	0.7107	0.5797	0.5199	0.7287	0.6069	0.6259
			5	0.7087	0.5767	0.5189	0.7314	0.6071	0.6249
Type II	7.06%	Multiple Imputation	1	0.8254	0.2634	0.2469	0.5172	0.3342	0.8545
			2	0.8254	0.2634	0.2480	0.5172	0.3352	0.8552
			3	0.8254	0.2634	0.2546	0.5080	0.3392	0.8603
			4	0.8254	0.2634	0.2551	0.5071	0.3394	0.8606
			5	0.8254	0.2634	0.2554	0.5021	0.3386	0.8615
		Fill with Median	1	0.8254	0.2634	0.2027	0.6949	0.3139	0.7855
			2	0.8254	0.2634	0.2027	0.6949	0.3138	0.7855
			3	0.8254	0.2634	0.2027	0.6949	0.3138	0.7855
			4	0.8258	0.2638	0.2196	0.6153	0.3237	0.7985
			5	0.8254	0.2634	0.2023	0.6915	0.3130	0.7857
		Fill with Group Mean	1	0.8254	0.2634	0.2308	0.5909	0.3319	0.8321
			2	0.8254	0.2634	0.2308	0.5909	0.3319	0.8321
			3	0.8254	0.2634	0.2308	0.5909	0.3319	0.8321
			4	0.8308	0.2710	0.2244	0.6337	0.3314	0.8195
			5	0.8254	0.2634	0.2308	0.5909	0.3320	0.8321

[1] Pop. ErrorRate is the true observed APACHE error rate in the entire population.
[2] AUROC is the Area Under the Receiver Operating Characteristic curve.
[3] AUPRC is the Area Under the Precision-Recall Curve.
[4] F-score $= 2 \times \frac{\text{Precision}\times\text{Recall}}{\text{Precision}+\text{Recall}}$
[5] Accuracy $= \frac{TP+TN}{TP+TN+FP+FN}$, where TP = true positive, TN = true negative, FP = false positive, and FN = false negative.

References

1. Friedman, J., Hastie, T. & Tibshirani, R. Regularization paths for generalized linear models via coordinate descent. *J. statistical software* **33**, 1, DOI: 10.18637/jss.v033.i01 (2010).

2. TeamRC. *R: A Language and Environment for Statistical Computing*. R Foundation for Statistical Computing, Vienna, Austria (2020).
